# The Association of Sarcopenia and Central Obesity with Mortality Risk in Patients with Chronic Kidney Disease – a 2-Year Observational Study

**DOI:** 10.1016/j.cdnut.2022.100014

**Published:** 2022-12-22

**Authors:** Helene Dahl, Hanne Rosendahl-Riise, Hans-Peter Marti, Jutta Dierkes

**Affiliations:** 1 Department of Clinical Medicine, University of Bergen, Norway; 2 Mohn Nutrition Research Laboratory, Department of Clinical Medicine, University of Bergen, Norway; 3 Department of Medicine, Haukeland University Hospital, Bergen, Norway; 4 Department of Medical Biochemistry and Pharmacology, Haukeland University Hospital, Bergen, Norway

**Keywords:** chronic kidney disease, nutritional status, mortality, sarcopenia, kidney transplantation, hemodialysis

## Abstract

**Background:**

Patients with chronic kidney disease (CKD) face numerous challenges regarding their nutritional status, including undernutrition, wasting, overweight, and obesity. However, there is a gap in the knowledge on the importance of nutritional status on the survival of CKD in patients along the spectrum of progression of CKD.

**Objectives:**

This study aimed to investigate the association of several nutritional measures with all-cause mortality. The hypothesis was that indicators of nutritional status exceeding BMI are associated with increased mortality risk.

**Methods:**

One-hundred seventy adult patients with predialysis CKD (*n* = 82), receiving hemodialysis (*n* = 42) or kidney transplantation (*n* = 46) were recruited from 2014 to 2019. At baseline, nutritional status was assessed by anthropometry, body composition, and muscle function by handgrip strength. Patient survival was assessed after a 2-y follow-up by Cox regression models adjusted for age, sex, and renal function and generalized additive models.

**Results:**

Thirty-one patients (18%) died during the 2-y follow-up. Sarcopenia (*n* = 30) was associated with an increased risk of death (HR: 2.92; 95% CI: 1.24, 6.89), whereas central obesity (*n* = 82) was not associated with mortality (1.05; 0.51, 2.15) in the Cox regression analyses. An association between BMI and mortality risk per unit increase (0.97; 0.90, 1.05) was not observed. Other markers of nutritional status were inversely associated with mortality risk, including handgrip strength (0.89; 0.83, 0.95), mid-upper arm circumference (0.86; 0.78, 0.95), and phase angle (per 0.1 degree increase 0.86; 0.81, 0.92). In the generalized additive models, U-shaped relationships were observed between mortality risk and waist circumference and mid-upper arm muscle circumference, while BMI < 22 kg/m^2^ was associated with increased mortality risk.

**Conclusions:**

Sarcopenia, but not central obesity was associated with total mortality in patients with CKD. The inclusion of muscle strength and mass measures in clinical practice should be considered.

## Introduction

The global burden of chronic kidney disease (CKD) has been increasing for the last decades, and it is predicted that the impact of CKD will continue to increase in the years to come [1, 2]. In 2017, it was estimated that 1.2 million people died from CKD, the 12th leading cause of death. This is an increase of 41.5% since 1990, and the global prevalence of CKD has increased by 30% since 1990. The world’s population has a longer life expectancy, and they also live longer with diseases such as CKD. It is projected that CKD will be the fifth leading cause of death in 2040, which underscores the magnitude of CKD and calls for further development of treatment for patients with CKD [2].

CKD comprises a heterogeneous group of diseases that embrace a variety of primary diseases that cause reduced functionality of the kidneys [3]. Patients with CKD often experience common consequences in terms of comorbidities, numerous prescribed medications, and changes in metabolism and nutritional status [[4], [5], [6]]. This may result in adverse changes in nutritional status, change in nutrient requirements, frequent dietary restrictions, and reduced physical activity levels [4].

Assessment of nutritional status of an individual reflects the dietary intake and utilization of nutrients and should be regularly performed in patients with CKD [7, 8]. Nutritional assessment comprehends information about dietary intake, biochemistry, anthropometry, as well as body composition, and function. Nutritional status can be expressed as reduced or increased weight, altered body shape (or distribution of adipose tissue), reduced muscle mass or function, deficiencies in micronutrients, and increased or reduced dietary intake. Particularly, nutritional risk or undernutrition is associated with more frequent hospital admissions, reduced capacity to endure medical treatment, increased mortality risk, and reduced quality of life [9]. In a clinical hospital setting, the most common assessment of nutritional status is the measurement of height and weight to calculate BMI as an indirect measure of body fat and obesity. This is a quick, easy, and cheap measurement to conduct and gives an estimate of the patient’s nutritional status. However, BMI does not reveal any information about body composition or body shape, nor information about body function. Body composition can give information on body fat and, even more in focus, skeletal muscle mass, which is needed to define sarcopenia [10, 11]. Sarcopenia also requires the measurement of muscle strength, while the distribution of fat mass is decisive for the definition of central obesity [12].

Sarcopenia is “a syndrome characterized by progressive and generalized loss of muscle mass and strength with a risk of adverse outcomes including physical disability, poor quality of life, and death” [11]. The etiology is not fully understood, but there are several factors in CKD that are believed to increase the risk of sarcopenia [13]. Even though the prevalence of sarcopenia in CKD has been investigated, numbers are highly divergent especially in predialysis CKD and in patients with kidney transplant [14]. Central obesity is associated with an increased risk of metabolic disease, cardiovascular disease (CVD), and mortality in the general population; however, the respective noxious evidence on patients with CKD is scarce, even though it is a common condition in CKD [[15], [16], [17]]. Moreover, there is a discussion on the disease-specific cut-off values for conditions such as sarcopenia and central obesity, and the analysis of the continuous measurements of nutritional status is warranted [[18], [19], [20]].

A thorough assessment of nutritional status conducted regularly is recommended by the National Kidney Foundation’s Kidney Disease Outcomes Quality Initiative (KDOQI) [8]. In a previous study, we observed a high prevalence of sarcopenia and central obesity in this population [21]. Indeed, studies have related mortality risk to nutritional status, particularly in patients receiving hemodialysis [22], but there is a lack of studies that have investigated the importance of nutritional status for mortality risk along the continuum of CKD, from early stages to after kidney transplantation. In the current study, the aim was to investigate the association between baseline indicators of nutritional status and total mortality during a follow-up time of 2 y. We hypothesize that sarcopenia and central obesity are related to mortality along the continuum of CKD patients, and further, measures of body composition and body shape would be more predictive of mortality than BMI.

## Methods

Adult, predominantly Caucasian, patients at different stages of CKD were included in this longitudinal observational study. The patients were recruited from November 2014 until December 2019 at Haukeland University Hospital, Bergen, Norway. Patients were eligible for inclusion if they had either an established CKD stage 3-5 before dialysis, end-stage kidney disease (ESKD) treated with stable hemodialysis or successful kidney transplantation. The patients had to be aged >18 y and be able to communicate in Norwegian or English. Patients with an estimated life expectancy of <6 mo were not considered for the study. The study was approved by the Regional Committee for Medical and Health Research of Western Norway (REK Vest) and conducted following the principles of the Helsinki Declaration. Written and informed consent was collected before study participation, which allowed future use of the collected data. The patients were not compensated for participation as such, but they were offered to receive feedback on their diet and other data collected, as well as a copy of the thesis associated with the data collection. The data set was always anonymized at a secure data server. The list of patient identification was kept separate from the data set and the principal investigator was responsible for the data security.

**FIGURE 1:**
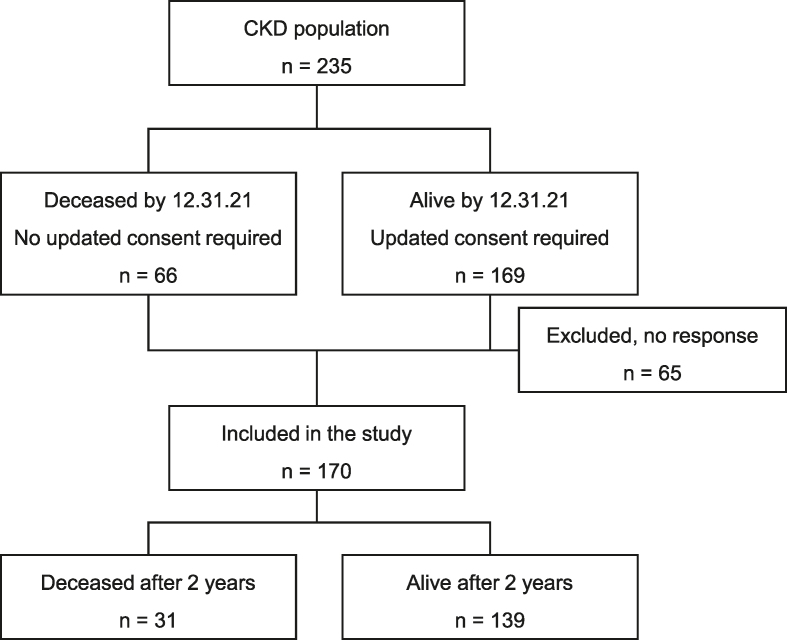
Flow chart of the inclusion process in the study. CKD, chronic kidney disease.

Follow-up time was similar for all patients and set to 2 y after inclusion, with no further contact between researchers and patients. An updated consent was required from the patients in the initial study population of 235 patients still alive by 31 December 2021. By the end of 2021, 66 patients were deceased, and out of the remaining 169 patients who were still alive, 104 provided written consent (mean follow-up time: 4.3 y). This resulted in 170 patients included in the study (Figure 1).

At baseline, kidney function was determined by the estimated glomerular filtration rate (eGFR) using the CKD-Epi equation based on creatinine measures [23]. CKD stages were classified by the eGFR following Kidney Disease – Improving Global Outcomes [3]. Additional information regarding routine blood analysis, prescribed medications, and comorbidities was retrieved from the patient record. Blood samples and measures were not taken in a fasting state, but in patients receiving hemodialysis, blood samples were taken before dialysis after the long weekend interval.

Nutritional status was thoroughly assessed at baseline, using measures of anthropometry, body composition, and functionality. Weight and height were measured using a Seca 877 weight (Seca) and Seca 217 stadiometer (Seca), respectively, wearing light clothes, and no shoes. Waist circumference and mid-upper arm circumference (MUAC) were measured on bare skin, with a flexible nonelastic tape. Measures of waist circumference were taken at the midpoint between the lower costal arch and the ileac crest in an outbreath position, and the mean of 3 measures was applied. MUAC was measured on the nondominant side, at the midpoint between the olecranon process of the ulna and the acromion process of the scapula. Skinfold triceps (SFT) measures were taken at the same position as MUAC by a Lange skinfold caliper (Quick Medical), and the mean measure of 3 measures was applied. Body composition was measured by a single frequency tetrapolar by bioelectrical impedance analysis (BIA) 101 Anniversary sport Edition (AKERN) device (after dialysis in patients receiving hemodialysis), while handgrip strength (HGS) was measured by a JAMAR hydraulic hand dynamometer (Sammons Preston). BIA was measured once, while HGS was measured 3 times at each arm. The max measure was applied, irrespective of hand dominance. Further measures were calculated; appendicular lean mass (ALM_BIA_) using the formula by MacDonald et al. [24], appendicular lean mass index (ALMI_BIA_), mid-upper arm muscle circumference (MUAMC), and the diagnosis of sarcopenia (EWGSOP2) [10] and central obesity (WHO) [25] was identified. Phase angle, which has been suggested as a measure of muscle quality, was also derived from BIA [10]. The patients were also screened for risk of undernutrition by nutritional risk screening 2002 (NRS2002) [26]. A detailed overview of the diagnosis criteria applied can be found in Table 1, while an overview of missing values for the respective measures can be found in Supplemental Table 1. Missing values were left out of the analyses.

**TABLE 1:** Diagnosis and respective diagnosis criteria applied in the study

Diagnosis	Criteria
**Nutritional status/exposure**	
Sarcopenia	Handgrip strength < 27/16 kg for male/female *and* Appendicular lean mass < 20/15 kg for male/female
Central obesity	Waist circumference ≥ 102/88 cm for male/female
NRS 2002	*Introductory screening:* Questions regarding low BMI, loss of weight, low dietary intake, and critical illness. If answered yes to at least one question, continuation to main screening. *Main screening:* Score within the categories of nutritional status, disease status, and age A total score of ≥3 indicates nutritional risk of undernutrition
**Comorbidities/Confounder**	
Diabetes	As stated in the patient record or Explicit diagnosis: E10-14 or HbA1c ≥ 48 mmol/mol or Prescription of antidiabetic medications: A10A-B
Cardiovascular disease	Diagnosis of coronary heart disease I20-25 or Diagnosis of atrial fibrillation I48 or Diagnosis of heart failure I50 or Diagnosis of total stroke I60-61, 63-64 except I63.6

Information about comorbidities was collected from the patient records. Diabetes was identified either by a diagnosis of diabetes in the patient record (written in text or given a diagnosis of ICD10 E10-14) at the time of inclusion, by HbA1c levels exceeding the diagnostic criteria for diabetes (≥48 mmol/mol) at the time of inclusion, or by prescription of antidiabetic medications (Anatomical Therapeutic Chemical Classifications system level 2, A10) [27]. CVD at baseline was defined by the registration of an ICD-10 diagnosis defined as coronary heart disease (I20-25), atrial fibrillation (I48), heart failure (I50), or total stroke (I60-I61, I63-I64 except for I63.6).

The cause of death was collected from the patient record when applicable. The causes were categorized as associated with CVD, CKD (with and without dialysis), cancer, infections/multiorgan failure, or unknown cause of death. The data collection of mortality status and cause of death was done by one investigator for all patients. The investigator collected the information systematically and did not have access to the information about nutritional status. Because of the routines regarding the death registry in Norway, the data collection on mortality status is regarded as complete in the current study.

### Statistical analysis

Patient characteristics are presented according to whether they were alive or deceased after 2 y. The groups are presented with means and SDs or counts and percentages. The association between nutritional status and mortality was investigated using Kaplan-Meier curves and Cox regression models for the diagnosis of sarcopenia and central obesity. Cox regression HRs were also estimated for HGS, ALM_BIA_, ALMI_BIA_, phase angle, waist circumference, skinfold triceps, MUAC, and MUAMC. The Cox regression models were adjusted for age and eGFR for all indicators and additionally adjusted for sex for the continuous markers. Additional models were created, where adjustments for serum albumin, diabetes, CVD, or dialysis treatment were added one by one to the original model. Generalized additive models (GAMs) were plotted for the association between markers of nutritional status as continuous variables and mortality risk to explore nonlinear relationships. Statistical analyses were performed using R software version 4.0.3 (The R Foundation for Statistical Computing, Vienna, Austria), and the packages within the *“Tidyverse”* [28]; *«dplyr»* [29], *«tidyr»* [30], *«ggplot2»* [31], and *«lubridate»* [32], as well as the packages “*Survival*” [33] and “*Survminer*” [34], and the function “*plotHR*” [35].

## Results

Among the 170 patients included, hypertensive and diabetic nephropathy was the primary cause of CKD causing 30% of the CKD cases, followed by glomerular diseases, which accounted for 19% of the cases. During the 2-y follow-up period, 31 patients (18%) died, of which the first patient died after 28 d. Among the 31 patients who died, 7 patients (23%) died of CKD (with and without dialysis), 6 (19%) died of CVD, 5 (16%) died of cancer, 4 (13%) died of infections and multiorgan failure, and in the remaining 9 (29%) patients the cause of death was unknown. None of the deaths was related to COVID-19.

During follow-up, 148 patients (87%) had the same treatment modality as that at baseline. Ten patients started dialysis during follow-up (8 from predialysis CKD stages 3-5 and 2 from kidney transplanted patients), and 10 patients received kidney transplantation (8 of the hemodialysis patients and 2 of the predialysis CKD stages 3-5 patients).

**FIGURE 2:**
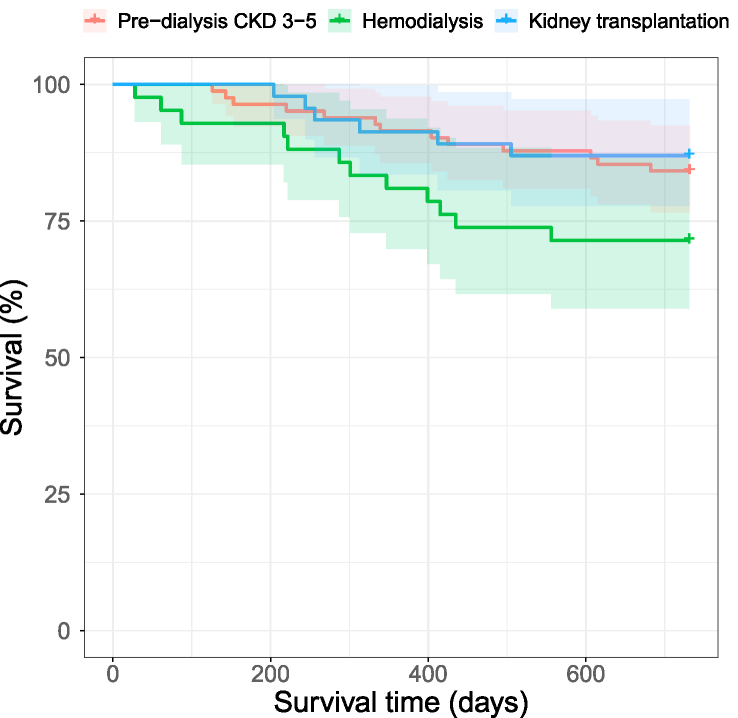
Kaplan-Meier curve of survival according to chronic kidney disease modality in the study population. CKD, chronic kidney disease.

An overview of characteristics of the study population at baseline according to mortality status after 2 y is shown in Table 2, and Supplemental Table 2 presents an overview of characteristics according to treatment modality. The patients who deceased after 2 y were older, had lower eGFR, and had a higher prevalence of diabetes. Patients receiving hemodialysis had the highest mortality rate, while survival was more similar among patients with predialysis stages 3-5 and kidney transplantation, as presented in Figure 2. The patients at CKD stage 3-5 before dialysis had the highest mean age.

**TABLE 2:** Baseline characteristics according to mortality status 2 y after inclusion

Variable	Alive, *n* = 139	Dead, *n* = 31
Female patients, *n* (%)	39 (28%)	9 (29%)
Age, y	61.0 (±14.6)	74.1 (±12.9)
Smoking, *n* (%)		
Never	53 (38.1%)	8 (25.8%)
Previous	71 (51.1%)	16 (51.6%)
Current	15 (10.8%)	7 (22.6%)
BP systolic, mmHg	138 (±19)	140 (±25)
BP diastolic, mmHg	76 (±11)	72 (±13)
CVD, *n* (%)	36 (26%)	13 (42%)
Hypertension, *n* (%)	85 (61%)	20 (65%)
Diabetes, *n* (%)	36 (26%)	14 (45.2%)
DM1	5 (4%)	4 (12.9%)
DM2	31 (22%)	10 (32.3%)
Number of prescribed medications	9 (±4)	14 (±5)
eGFR, mL/min/1.73 m^2^	30 (±21)	20 (±18)
Creatinine, μmol/L	329 (±286)	403 (±242)
CRP, mg/L	5.2 (±13.3)	13.1 (±21.7)
Albumin, g/L	43.1 (±3.2)	39.5 (±4.2)
Hemoglobin, g/dL		
Male	13.1 (± 2.0)	12.5 (± 1.4)
Female	11.9 (±1.4)	11.2 (±1.9)
HbA1c, mmol/mol	40.4 (±9.8)	46.4 (±12.5)

BP, blood pressure; eGFR, estimated glomerular filtration rate; CVD, cardiovascular disease; DM1/2, diabetes mellitus type 1/2

Numbers are presented as means (SD) or counts (percentage).

Dialysis quality and vintage in patients receiving hemodialysis and time since transplantation in kidney transplant patients were not related to mortality, respectively. There was a higher mean number of prescribed medications among deceased patients compared with the patients who were alive after 2 y. We also observed a higher prevalence of current smokers among the deceased patients, while the prevalence of never-smokers was higher among the patients alive.

Characteristics of nutritional status at baseline according to mortality status after 2 y of follow-up are presented in Table 3, and Supplemental Table 3 gives an overview of nutritional status according to treatment modality. The prevalence of sarcopenia was higher among deceased patients, while the prevalence of central obesity was not different. There was no difference in either waist circumference or BMI. In contrast, we observed differences in HGS, ALM_BIA_, phase angle, and MUAC. The overall prevalence of nutritional risk was low (9%). Nevertheless, there was a higher prevalence of patients at nutritional risk among deceased patients (23%) compared with patients alive (7%). The kidney transplanted patients had a lower prevalence of sarcopenia (6.5%) compared with the other groups (22% and 21.4%), and the CKD patients in stages 3-5 before dialysis had the highest prevalence of central obesity (54.9% vs. 45.7% and 38.1%).

**TABLE 3:** Baseline characteristics of nutritional status according to mortality status after 2 y of follow-up

Variable	Alive, *n* = 139	Dead, *n* = 31
BMI, kg/m^2^	26.6 (±4.5)	26.0 (±4.9)
Central obesity, *n* (%)	67 (48%)	15 (48%)
Waist circumference, cm		
Male	101 (± 13)	99 (± 14)
Female	94 (±15)	89 (±11)
Sarcopenia, *n* (%)	14 (10%)	16 (52%)
HGS, kg		
Male	37 (± 11)	25 (± 6)
Female	23 (± 7)	13 (±4)
ALM_BIA_, kg		
Male	24.1 (± 5.1)	20.1 (± 3.2)
Female	15.4 (±2.4)	13.1 (±2.5)
ALMI_BIA_, kg/m^2^		
Male	7.7 (±1.2)	6.9 (±0.9)
Female	5.8 (±0.7)	5.2 (±1.1)
Phase angle, °		
Male	5.7 (±1.2)	4.4 (±0.8)
Female	5.5 (±0.9)	3.6 (±1.0)
SFT, mm		
Male	20 (±9)	18 (±8)
Female	27 (±8)	18 (±7)
MUAC, cm		
Male	31.6 (±4.2)	29.9 (±4.2)
Female	31.2 (±4.0)	26.0 (±3.2)
MUAMC, cm		
Male	25.7 (±3.1)	25.2 (±3.8)
Female	23.3 (±3.2)	21.4 (±3.2)

ALM_BIA_, appendicular lean mass assessed by bioelectrical impedance analysis; ALMI_BIA_, appendicular lean mass index assessed by bioelectrical impedance analysis; HGS, handgrip strength; MUAC, mid-upper arm circumference; MUAMA, mid-upper arm muscle area; MUAMC, mid-upper arm muscle circumference; SFT, skinfold triceps.

Numbers are presented as means (SD) or counts (percentage). Diagnosis of central obesity was given when waist circumference exceeded measures of 102 and 88 cm for males and females, respectively. Sarcopenia is defined according to the revised consensus from the European Working Group on Sarcopenia in Older People [10].

**FIGURE 3:**
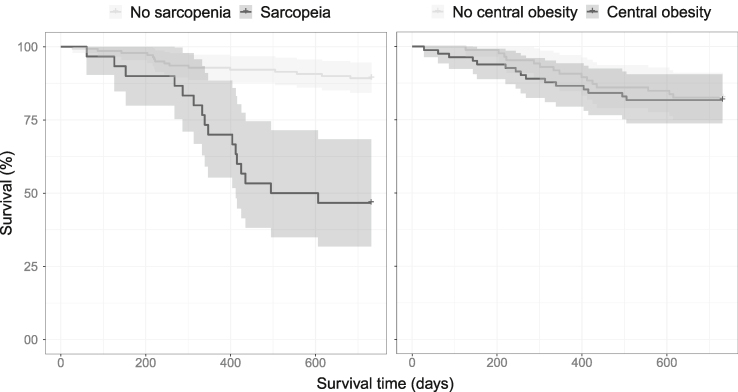
Kaplan-Meier survival curves for the study population according to the diagnosis of either sarcopenia (EWGSOP2) or central obesity (WHO).

The survival times across diagnoses of sarcopenia and central obesity are presented as Kaplan-Meier plots in Figure 3. Patients with sarcopenia showed higher mortality after approximately 300 d, and the difference in mortality rate continued to increase throughout the follow-up. The survival probability was similar in patients with and without central obesity.

In the Cox regression models with adjustments for age and eGFR, we observed similar results, with increased mortality risk associated with the diagnosis of sarcopenia (HR: 2.92; 95% CI: 1.24, 6.89) but not with central obesity (1.05; 0.51, 2.15). Additional one by one adjustments for serum albumin, diabetes, CVD, or treatment with dialysis did not change the estimated risk substantially (Supplemental Table 4).

**FIGURE 4:**
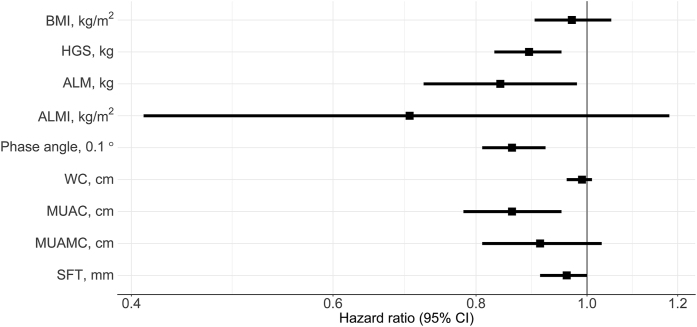
HRs for mortality according to measures of nutritional status, from Cox regression analysis adjusted for age, sex and eGFR. HRs per unit increase if not otherwise stated.

Measures of nutritional status (continuous variables) in Cox regression models adjusted for age, eGFR, and sex, are shown in Figure 4. All measures were either negatively associated with increased mortality risk or not associated with it. We observed a decreased risk of mortality per increased kg of HGS (0.88; 0.83, 0.94), per cm increase in MUAC (0.87; 0.78, 0.96), and per 0.1 degree increase in phase angle (0.86; 0.81, 0.92). No clear associations were observed per unit (kg/m^2^) increase in BMI (0.98; 0.90, 1.07) and per cm increase in waist circumference (0.99; 0.96, 1.02). Additional one-by-one adjustments for serum albumin, CVD, diabetes, or dialysis treatment did not change the observed associations (Supplemental Table 5). Additional adjustments for inflammation and smoking were performed; however, this did not change the observed association between nutritional status and mortality risk in this study (data not shown).

**FIGURE 5:**
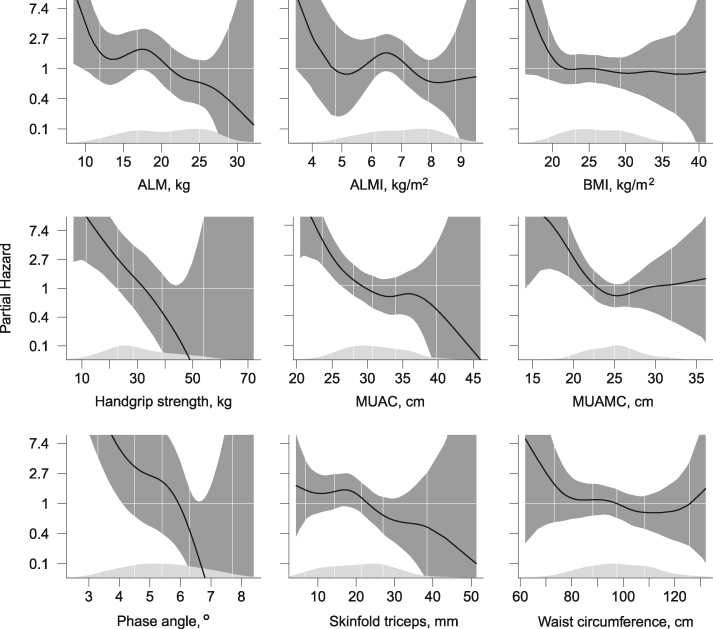
Generalized additive models for the Cox regression models adjusted for age, sex and estimated glomerular filtration rate (eGFR). The dark gray areas around the central line indicate the 95% CIs of the hazard estimates. The light gray areas at the x-axis are the density plots of each measure of nutritional status, and the vertical lines represent the percentiles of 2.5, 25, 50, 75, and 97.5.

GAM analyses of the relationship of continuous variables with mortality are presented in Figure 5. We observed a negative linear relationship between mortality risk and measures of ALM_BIA_, HGS, MUAC, phase angle, and SFT. Measures of BMI showed no relationship to mortality risk, apart from BMI < 22 kg/m^2^, for which we observed a negative linear relationship with mortality. For waist circumference and MUAMC, we observed a U-shaped relationship with increased mortality risk with extreme values.

## Discussion

In this study, we investigated the association between nutritional status and mortality risk in CKD patients after 2 y of follow-up using a wide range of measures of nutritional status. Sarcopenia was associated with an increased risk of mortality, while central obesity was not associated with mortality. Regarding the continuous markers of nutritional status, inverse associations with mortality were observed for HGS, ALM_BIA_, phase angle, and MUAC. As HGS and ALM_BIA_ are the measures comprising the sarcopenia diagnosis, this was not a surprise. Additionally, phase angle, the ratio between the resistance and reactance from the BIA, has also been proposed as an alternative measure of muscle quality by EWGSOP [10]. Results from the present study show that a low phase angle was associated with higher mortality risk and could be a useful indicator of nutritional status. There was no obvious mortality risk associated with BMI, ALMI_BIA_, MUAMC, and waist circumference. When investigating nonlinear relationships, we found a U-shaped association between mortality risk and both MUAMC and waist circumference, while BMI was only associated with increased risk at BMI <22 kg/m^2^; however, the estimates observed include large uncertainty (Figure 5). Measures that are traditionally associated with increased mortality risk such as inflammation, low serum albumin, and smoking were also associated with mortality in our study; however, adjustment for these factors did not change the observed associations between mortality risk and nutritional status [36, 37].

The findings from our study demonstrate that a variety of measurements may be necessary to assess nutritional status, beyond the measurements of weight and height. Even though in clinical practice, the assessment of nutritional status is often focused on the measurements of height and weight, and the calculation of BMI, it has to be taken into account that a high BMI does not preclude diagnoses of malnutrition and sarcopenia, as shown by us [21], and others [9, 38]. BMI is not able to capture the body composition, body shape, or functionality of an individual, nor the dietary intake, and thus is not sufficient in the assessment of nutritional status.

A common trait of the indicators of nutritional status associated with mortality in our study was the relation to muscle mass and strength, which has also been reported in other studies [22]. This suggests that muscle health is important for survival in patients with CKD, as it is in the general population [10]. Today, there are discrepancies in the diagnosis criteria for sarcopenia, and the prevalence of sarcopenia will vary according to which definition is applied [39, 40]. In 2016, sarcopenia was introduced in the ICD-10 code system; however, the lack of a uniform definition of sarcopenia is reflected in a low prevalence of coding of sarcopenia in the patient records [41, 42]. In an extensive study of sarcopenia, obesity, and mortality in a US population, sarcopenia (defined as low skeletal muscle mass index without measurement of muscle strength) was associated with increased mortality, irrespective of kidney function [43]. In another large study from the UK, most analyses on sarcopenia and mortality risk were performed on probable sarcopenia (defined as low HGS without any confirmation of low muscle mass). These examples demonstrate the challenge of the lack of common diagnostic criteria for sarcopenia [41]. It is also questioned whether disease-specific criteria for sarcopenia should be defined [39].

To date, the reasons for the exaggerated rates of sarcopenia in CKD have not been fully elucidated [41]. Sarcopenia may be age-related (primary sarcopenia) or related to other factors, such as chronic disease (secondary sarcopenia) [10]. As CKD is more prevalent by age, the concordance of the conditions may be partly driven by aging (primary sarcopenia). However, CKD is also considered a model of accelerated aging, exacerbated by the nature of the CKD in terms of accumulation of uremic toxins, inflammation, oxidative stress, insulin resistance, and a low dietary intake of energy and proteins [13, 41, 44]. Consequently, a negative nitrogen balance may occur, causing the breakdown of muscle tissue [13]. Low physical activity levels are also associated with CKD, which will further contribute to sarcopenia [45]. Such determinants are also associated with the progression of CKD and mortality, and it remains to be investigated whether there is a causal relationship between either muscle strength and muscle mass or with mortality or whether this relationship is purely associative in patients with CKD.

We observed no association between central obesity and mortality, which is in line with the obesity paradox, which is frequently described in the CKD population in the literature [46]. It is suggested that patients with excess weight and waist circumference may be resistant to the weight loss related to illness and inflammation which is on the other hand associated with increased mortality [46]. Because of the limited sample size, we did not have the opportunity to combine the diagnoses of central obesity and sarcopenia, which would make it possible to distinguish between excess fat mass with and without muscle loss. Additionally, as this was an observational study with only one baseline measurement of nutritional status it is not possible to draw any causal conclusion from the current study. Studies have shown that lifestyle interventions to promote weight loss associated may slow the progression of CKD, as well as improvement of kidney function in predialysis CKD [17]. The long-term effects of lifestyle interventions and more research on other modalities of CKD are still required.

In this study, we have included a large variety of patients with CKD, from early stages (CKD stage 3) to after transplantation. To our knowledge, such a study has not been performed in such a varied group of CKD patients before. In our population, the patients treated with hemodialysis at baseline had the highest mortality risk after 2 y of follow-up; however, when adjusting for dialysis treatment, our results did not change (Supplemental Table 4). We also included a wide range of measures of nutritional status, allowing us to assess nutritional status thoroughly. However, further separated analyses according to treatment modality were not performed because of a rather low number of participants. The findings in the present study would be interesting to investigate in a larger study, allowing analyses stratified by treatment modality and CKD stage.

There are also some limitations to be considered. This was an observational study, and we only assessed nutritional status at baseline. Due to the requirement of another written consent to include patients after 2 y of follow-up, 69/235 of the original study population was lost to follow-up. This would cause a false high mortality rate in the study group when analyzed without considering this. Even though our assessment of nutritional status was thorough, we did not include an assessment of dietary intake, physical activity, or physical function. In addition, our measurement of body composition by BIA may be influenced by hydration status; however, the measurement was performed in a standardized way in all patients and after dialysis treatment in hemodialysis patients. Even though the regression models were adjusted for relevant factors, a fully adjusted Cox regression analysis was not performed due to the relatively low mortality rate in the study population.

In the present study, we have found an association between indicators of nutritional status and mortality risk, especially markers related to muscle health. Markers such as HGS and MUAC can identify different aspects of nutritional status compared with BMI, and the results suggest that implementation of such measures in the clinical setting would be of importance to identify patients at increased mortality risk. More studies are required to confirm this association. Future studies should investigate whether dietary and lifestyle interventions can improve indicators of nutritional status and thus improve mortality risk, and overall health in these patients.

## Author disclosures

The authors report no conflicts of interest.

## Data Availability

Data described in the manuscript, code book, and analytic code will be made available upon request.

## Appendix A Supplementary data

Multimedia components 1 Multimedia components 1

## References

[1] Bikbov B., Purcell C.A., Levey A.S., Smith M., Abdoli A., Abebe M. (2020). Global, regional, and national burden of chronic kidney disease, 1990–2017: A systematic analysis for the Global Burden of Disease Study 2017. Lancet.

[2] Foreman K.J., Marquez N., Dolgert A., Fukutaki K., Fullman N., McGaughey M. (2018). Forecasting life expectancy, years of life lost, and all-cause and cause-specific mortality for 250 causes of death: reference and alternative scenarios for 2016–40 for 195 countries and territories. Lancet.

[3] Kidney Disease: Improving Global Outcomes, CKD Work Group (2013). KDIGO 2012 clinical practice guideline for the evaluation and management of chronic kidney disease. Kidney Int Suppl.

[4] Hendriks F.K., Kooman J.P., van Loon L.J.C. (2021). Dietary protein interventions to improve nutritional status in end-stage renal disease patients undergoing hemodialysis. Curr Opin Clin Nutr Metab Care.

[5] Kalantar-Zadeh K., Jafar T.H., Nitsch D., Neuen B.L., Perkovic V. (2021). Chronic kidney disease. Lancet.

[6] Dahl H., Sandblost S.R.T., Welland N.L., Sandnes K., Sekse I., Sæle K. (2021). Medication prescription, common side-effects, and nutritional status are associated in patients with chronic kidney disease. J Ren Nutr.

[7] Cederholm T., Barazzoni R., Austin P., Ballmer P., Biolo G., Bischoff S.C. (2017). ESPEN guidelines on definitions and terminology of clinical nutrition. Clin Nutr.

[8] Ikizler T.A., Burrowes J.D., Byham-gray L.D., Campbell K.L., Carrero J., Chan W. (2020). KDOQI clinical practice guideline for nutrition in CKD: 2020 update. Am J Kidney Dis.

[9] Tangvik R.J., Tell G.S., Guttormsen A.B., Eisman J.A., Henriksen A., Nilsen R.M. (2015). Nutritional risk profile in a university hospital population. Clin Nutr.

[10] Cruz-Jentoft A.J., Bahat G., Bauer J., Boirie Y., Bruyere O., Cederholm T. (2019). Sarcopenia: revised European consensus on definition and diagnosis. Age Ageing.

[11] Cruz-Jentoft A.J., Baeyens J.P., Bauer J.M., Boirie Y., Cederholm T., Landi F. (2010). Sarcopenia: European consensus on definition and diagnosis: Report of the European Working Group on Sarcopenia in Older People. Age Ageing.

[12] World Health Organisation (2008). http://www.who.int.

[13] Sabatino A., Cuppari L., Stenvinkel P., Lindholm B., Avesani C.M. (2021). Sarcopenia in chronic kidney disease: what have we learned so far?. J Nephrol.

[14] Shu X., Lin T., Wang H., Zhao Y., Jiang T., Peng X. (2022). Diagnosis, prevalence, and mortality of sarcopenia in dialysis patients: a systematic review and meta-analysis. J Cachexia Sarcopenia Muscle.

[15] Conley M.M., McFarlane C.M., Johnson D.W., Kelly J.T., Campbell K.L., MacLaughlin H.L. (2021). Interventions for weight loss in people with chronic kidney disease who are overweight or obese. Cochrane Database Syst Rev.

[16] Ladhani M., Craig J.C., Irving M., Clayton P.A., Wong G. (2017). Obesity and the risk of cardiovascular and all-cause mortality in chronic kidney disease: A systematic review and meta-analysis. Nephrol Dial Transplant.

[17] Chintam K., Chang A.R. (2021). Strategies to treat obesity in patients with CKD. Am J Kidney Dis.

[18] Agarwal R., Bills J.E., Light R.P. (2010). Diagnosing obesity by body mass index in chronic kidney disease: An explanation for the “obesity paradox?”. Hypertension.

[19] Dai L., Mukai H., Lindholm B., Heimbürger O., Barany P., Stenvinkel P. (2017). Clinical global assessment of nutritional status as predictor of mortality in chronic kidney disease patients. PLoS One.

[20] Davis E., Campbell K., Gobe G., Hawley C., Isbel N., Johnson D.W. (2016). Association of anthropometric measures with kidney disease progression and mortality: a retrospective cohort study of pre-dialysis chronic kidney disease patients referred to a specialist renal service. BMC Nephrol.

[21] Dierkes J., Dahl H., Welland N.L., Sandnes K., Saele K., Sekse I. (2018). High rates of central obesity and sarcopenia in CKD irrespective of renal replacement therapy - an observational cross-sectional study. BMC Nephrol.

[22] Ribeiro H.S., Neri S.G.R., Oliveira J.S., Bennett P.N., Viana J.L., Lima R.M. (2022). Association between sarcopenia and clinical outcomes in chronic kidney disease patients: A systematic review and meta-analysis. Clin Nutr.

[23] Levey A.S., Stevens L.A., Schmid C.H., Zhang Y.L., Castro A.F., Feldman H.I. (2009). A new equation to estimate glomerular filtration rate. Ann Intern Med.

[24] Macdonald J.H., Marcora S.M., Jibani M., Roberts G., Kumwenda M.J., Glover R. (2006). Bioelectrical impedance can be used to predict muscle mass and hence improve estimation of glomerular filtration rate in nondiabetic patients with chronic kidney disease. Nephrol Dial Transpl.

[25] Lean M.E.J., Han T.S., Morrison C.E. (1995). Waist circumference as a measure for indicating need for weight management [Internet]. BMJ.

[26] Kondrup J., Rasmussen H.H., Hamberg O., Stanga Z. (2003).

[27] WHO Collaborating Centre for Drug Statistics Methodology ATC: Structure and principles. https://www.whocc.no/atc/structure_and_principles/.

[28] Wickham H., Averick M., Bryan J., Chang W., McGowan L.D., Francois R. (2019). Tidyverse: easily install and load the “tidyverse. J Open Source Softw [Internet].

[29] Wickham H., Francois R., Henry L., Müller K. (2021). https://cran.r-project.org/package=dplyr.

[30] Wickham H. (2021). https://cran.r-project.org/package=tidyr.

[31] H Wickham, ggplot2: Elegant Graphics for Data Analysis, Springer-Verlag, New York, ISBN 978-3-319-24277-4, 2016. https://ggplot2.tidyverse.org.

[32] Grolemund G., Wickham H. (2011). Dates and times made easy with lubridate. J Stat Softw.

[33] Therneau T. (2021). https://cran.r-project.org/package=survival.

[34] Kassambara A., Kosinski M., Biecek P. (2021). https://cran.r-project.org/package=survminer.

[35] Seifert R. (2009). http://rforge.org/2009/10/30/plot-function-for-additive-cox-proportional-hazard-regression/.

[36] Fouque D., Kalantar-Zadeh K., Kopple J., Cano N., Chauveau P., Cuppari L. (2008). A proposed nomenclature and diagnostic criteria for protein-energy wasting in acute and chronic kidney disease. Kidney Int.

[37] Fiedler R., Jehle P.M., Osten B., Dorligschaw O., Girndt M. (2009). Clinical nutrition scores are superior for the prognosis of haemodialysis patients compared to lab markers and bioelectrical impedance. Nephrol Dial Transplant.

[38] Ng W.L., Collins P.F., Hickling D.F., Bell J.J. (2019). Evaluating the concurrent validity of body mass index (BMI) in the identification of malnutrition in older hospital inpatients. Clin Nutr.

[39] Bellafronte N.T., Sizoto G.R., Vega-Piris L., Chiarello P.G., Cuadrado G.B. (2020). Bed-side measures for diagnosis of low muscle mass, sarcopenia, obesity, and sarcopenic obesity in patients with chronic kidney disease under nondialysis-dependent, dialysis dependent and kidney transplant therapy. PLoS One.

[40] Fernandes L.V., Paiva A.E.G., Silva A.C.B., de Castro I.C., Santiago A.F., de Oliveira E.P. (2022). Prevalence of sarcopenia according to EWGSOP1 and EWGSOP2 in older adults and their associations with unfavorable health outcomes: a systematic review. Aging Clin Exp Res.

[41] Wilkinson T.J., Miksza J., Yates T., Lightfoot C.J., Baker L.A., Watson E.L. (2021). Association of sarcopenia with mortality and end-stage renal disease in those with chronic kidney disease: a UK Biobank study. J Cachexia Sarcopenia Muscle.

[42] Anker S.D., Morley J.E., von Haehling S. (2016). Welcome to the ICD-10 code for sarcopenia. J Cachexia Sarcopenia Muscle.

[43] Androga L., Sharma D., Amodu A., Abramowitz M.K. (2017). Sarcopenia, obesity, and mortality in US adults with and without chronic kidney disease. Kidney Int Rep.

[44] Roshanravan B., Gamboa J., Wilund K. (2017). Exercise and CKD: skeletal muscle dysfunction and practical application of exercise to prevent and treat physical impairments in CKD. Am J Kidney Dis.

[45] Hirai K., Ookawara S., Morishita Y. (2016). Sarcopenia and physical inactivity in patients with chronic kidney disease. Nephrourol Mon.

[46] Ziolkowski S.L., Long J., Baker J.F., Chertow G.M., Leonard M.B. (2019). Chronic kidney disease and the adiposity paradox: valid or confounded?. J Ren Nutr.

