# Supplementary material for: The Association of Sarcopenia and Central Obesity with Mortality Risk in Patients with Chronic Kidney Disease – a 2-Year Observational Study
Source: Curr Dev Nutr. 2022 Dec 22;7(1):100014. doi: 10.1016/j.cdnut.2022.100014 (PMC10100932; doi:10.1016/j.cdnut.2022.100014)
Supplement: Multimedia components 1 [file mmc1.docx]

**Supplemental material:**

***The association of sarcopenia and central obesity with mortality risk in patients with chronic kidney disease – a two-year observational study (Helene Dahl et al.)***

**Content:**

Supplemental Table 1: Missing values of included measures

Supplemental Table 2: Baseline characteristics according to treatment modality at baseline

Supplemental Table 3: Baseline characteristics of nutritional status according to treatment modality at baseline

Supplemental Table 4: Hazard Ratios and confidence intervals of mortality risk from Cox regression models of categorical markers of nutritional status

Supplemental Table 5: Hazard Ratios and confidence intervals of mortality risk from Cox regression models of continuous markers of nutritional status

**Supplemental Table 1: Missing values of included measures**

| **Measure** | **Total, n = 170** | **Alive, n = 139** | **Deceased, n = 31** |
| --- | --- | --- | --- |
| **Smoking** | 0 | 0 | 0 |
| **BP systolic** | 5 | 4 | 1 |
| **BP diastolic** | 5 | 4 | 1 |
| **CVD** | 0 | 0 | 0 |
| **Hypertension** | 0 | 0 | 0 |
| **Diabetes** | 0 | 0 | 0 |
| **No of medications** | 0 | 0 | 0 |
| **eGFR** | 0 | 0 | 0 |
| **Creatinine** | 2 | 2 | 0 |
| **CRP** | 6 | 6 | 0 |
| **Albumin** | 5 | 5 | 1 |
| **Haemoglobin** | 4 | 4 | 0 |
| **HbA1c** | 29 | 26 | 3 |
| **BMI** | 1 | 1 | 0 |
| **Central obesity** | 2 | 1 | 1 |
| **Waist circumference** | 2 | 1 | 1 |
| **Sarcopenia** | 0 | 0 | 0 |
| **HGS** | 1 | 0 | 1 |
| **ALM_BIA_** | 19 | 13 | 6 |
| **ALMI_BIA_** | 19 | 13 | 6 |
| **Phase angle** | 14 | 10 | 4 |
| **SFT** | 0 | 0 | 0 |
| **MUAC** | 0 | 0 | 0 |
| **MUAMC** | 0 | 0 | 0 |

ALM**_BIA_**, appendicular lean mass assessed by bioelectrical impedance analysis; ALMI**_BIA_**, appendicular lean mass index assessed by bioelectrical impedance analysis; BMI, body mass index; BP, blood pressure; CRP, c-reactive protein; CVD, cardiovascular disease; eGFR, estimated glomerular filtration rate; HGS, handgrip strength; MUAC, mid-upper arm circumference; MUAMC, mid-upper arm muscle circumference; SFT, skinfold triceps.

**Supplemental Table 2: Baseline characteristics according to treatment modality at baseline**

| **Variable** | **CKD, n = 82** | **HD, n = 42** | **KTR, n = 46** |
| --- | --- | --- | --- |
| **Deceased patients, n** | 13 (16%) | 12 (29%) | 6 (13%) |
| **Female patients, n** | 24 (29%) | 13 (31%) | 11 (24%) |
| **Age, years** | 66.0 (±14.6) | 61.2 (±18.5) | 60.7 (±12.0) |
| **Smoking, n** |  |  |  |
| **Never** | 27 (32.9%) | 13 (31.0%) | 21 (45.7%) |
| **Previous** | 43 (52.4%) | 23 (54.8%) | 21 (45.7%) |
| **Current** | 12 (14.6 %) | 6 (14.3%) | 4 (8.7%) |
| **BP systolic, mmHg** | 138.2 (±16.1) | 148.1 (±28.0) | 130.7 (1±3.8) |
| **BP diastolic, mmHg** | 77.1 (±10.5) | 70 (±15.9) | 77.3 (±7.5) |
| **CVD, n** | 30 (36.6%) | 23 (54.8%) | 19 (41.3%) |
| **Hypertension, n** | 55 (67.1%) | 34 (81.0%) | 16 (34.8%) |
| **Diabetes, n** | 25 (30.5%) | 16 (38.1%) | 8 (17.4%) |
| **DM1** | 3 (3.7%) | 5 (11.9%) | 1 (2.2%) |
| **DM2** | 22 (26.8%) | 11 (26.2%) | 7 (15.2%) |
| **Number of prescribed medications** | 7.2 (±3.6) | 15.5 (±3.8) | 9.4 (±3.3) |
| **eGFR, mL/min/1.73 m^2^** | 26.7 (±11.9) | 6.7 (±3.3) | 50 (±21.8) |
| **Creatinine, µmol/L** | 247 (±123) | 742.4 (±244) | 149 (±67) |
| **CRP, mg/L** | 6.5 (±14.0) | 10.8 (±23.3) | 3.2 (±3.1) |
| **Albumin, g/L** | 43.3 (±3.4) | 40.4 (±3.8) | 43.0 (±3.3) |
| **Haemoglobin, g/dL** |  |  |  |
| **Male** | 13.3 (±1.6) | 11.4 (±1.2) | 13.8 (±2.2) |
| **Female** | 11.8 (±1.2) | 10.8 (±1.3) | 13.1 (±1.4) |
| **HbA1c, mmol/mol** | 42.6 (±10.1) | 39.6 (±13.5) | 41.3 (±9.3) |
| BP, blood pressure; eGFR, estimated glomerular filtration rate; CRP, C-reactive protein; CVD, cardiovascular disease; DM1/2 diabetes mellitus type 1/2  Numbers are presented as means (standard deviation) or counts (percentage). | | | |

**Supplemental Table 3: Baseline characteristics of nutritional status according to treatment modality at baseline**

| **Variable** | **CKD, n = 82** | **HD, n = 42** | **KTR, n = 46** |
| --- | --- | --- | --- |
| **BMI, kg/m^2^** | 27.8 (±4.7) | 24.3 (±3.9) | 26.2 (±3.9) |
| **Central obesity, n** | 45 (54.9%) | 16 (38.1%) | 21 (45.7%) |
| **Waist circumference, cm** |  |  |  |
| **Male** | 102 (±13) | 97 (±14) | 100 (±13) |
| **Female** | 96 (±13) | 88 (±17) | 94 (±11) |
| **Sarcopenia, n** | 18 (22.0%) | 9 (21.4%) | 3 (6.5%) |
| **HGS, kg** |  |  |  |
| **Male** | 36 (±12) | 31 (±9) | 36 (±10) |
| **Female** | 21 (±10) | 21 (±6) | 22 (±4) |
| **ALM_BIA_, kg** |  |  |  |
| **Male** | 23.3 (±3.7) | 21.3 (±3.8) | 24.2 (±3.0) |
| **Female** | 15.3 (±2.6) | 13.9 (±3.1) | 15.6 (±1.5) |
| **ALMI_BIA_, kg/m^2^** |  |  |  |
| **Male** | 7.6 (±0.9) | 7.0 (±0.8) | 7.7 (±0.7) |
| **Female** | 5.8 (±0.8) | 5.3 (±1.0) | 5.8 (±0.4) |
| **Phase angle, °** |  |  |  |
| **Male** | 5.6 (±1.3) | 5.0 (±1.3) | 5.7 (±1.1) |
| **Female** | 5.3 (±1.0) | 4.4 (±1.3) | 5.7 (±0.9) |
| **SFT, mm** |  |  |  |
| **Male** | 22 (±8) | 18 (±10) | 17 (±8) |
| **Female** | 28 (±9) | 22 (±8) | 22 (±8) |
| **MUAC, cm** |  |  |  |
| **Male** | 31.5 (±4.7) | 29.8 (±4.1) | 30.6 (±3.0) |
| **Female** | 31.8 (±4.6) | 27.7 (±4.0) | 29.7 (±2.5) |
| **MUAMC, cm** |  |  |  |
| **Male** | 25.6 (±3.3) | 26.1 (±4.0) | 25.3 (±2.3) |
| **Female** | 22.9 (±2.5) | 23.3 (±5.2) | 22.7 (±1.8) |
| ALM**_BIA_**, appendicular lean mass assessed by bioelectrical impedance analysis; ALMI**_BIA_**, appendicular lean mass index assessed by bioelectrical impedance analysis; HGS, handgrip strength; MUAC, mid-upper arm circumference; MUAMA, mid-upper arm muscle area; MUAMC, mid-upper arm muscle circumference; SFT, skinfold triceps.  Numbers are presented as means (standard deviation) or counts (percentage). Diagnosis of central obesity was given when waist circumference exceeded measures of 102 and 88 cm for males and females, respectively. Sarcopenia is defined according to the revised consensus from the European Working Group on Sarcopenia in Older People (10). | | | |

**Supplemental Table 4: Hazard Ratios and confidence intervals of mortality risk from Cox regression models of categorical markers of nutritional status**

|  | **Model 1:** Age, eGFR | **Model 2:** Age, eGFR, albumin | **Model 3:** Age, eGFR, diabetes | **Model 4:** Age, eGFR, CVD | **Model 5:** Age, eGFR,  dialysis |
| --- | --- | --- | --- | --- | --- |
| **Sarcopenia** | 2.92  (1.24-6.89) | 2.67  (1.11-6.41) | 3.37  (1.4-8.09) | 2.90  (1.26-6.67) | 2.91  (1.23-6.92) |
| **Central obesity** | 1.05  (0.51-2.15) | 1.15  (0.55-2.39) | 0.97  (0.47-2.02) | 1.23  (0.59-2.56) | 1.12  (0.51-2.30) |

eGFR, estimated glomerular filtration rate. Cardiovascular disease consists of coronary heart disease (ICD10 I20-25), atrial fibrillation (ICD10 I48), heart failure (ICD10 I50), or total stroke (ICD10 I60-61 and I63-64, except I63.6).

**Supplemental Table 5: Hazard Ratios and confidence intervals of mortality risk from Cox regression models of continuous markers of nutritional status**

|  | **Model 1:** Age, sex, eGFR | **Model 2:** Age, sex, eGFR, albumin | **Model 3:** Age, sex, eGFR, diabetes | **Model 4:** Age, sex, eGFR, CVD | **Model 5:** Age, sex, eGFR, dialysis |
| --- | --- | --- | --- | --- | --- |
| **BMI, kg/m^2^** | 0.97  (0.90-1.05) | 0.98  (0.90-1.06) | 0.95  (0.87-1.04) | 0.98  (0.90-1.07) | 0.98  (0.90-1.07) |
| **HGS, kg** | 0.89  (0.83-0.95) | 0.90  (0.84-0.96) | 0.89  (0.83-0.95) | 0.89  (0.83-0.95) | 0.88  (0.83-0.94) |
| **ALM_BIA_, kg** | 0.84  (0.72- 0.98) | 0.83  (0.70-0.99) | 0.81  (0.69-0.96) | 0.87  (0.73-1.01) | 0.86  (0.73-1.02) |
| **ALMI_BIA_, kg/m^2^** | 0.70  (0.41-1.18) | 0.69  (0.41-1.17) | 0.62  (0.35-1.08) | 0.76  (0.43-1.27) | 0.82  (0.46-1.44) |
| **PA, 0.1 °** | 0.86  (0.81-0.92) | 0.90  (0.84-0.96) | 0.86  (0.81-0.92) | 0.87  (0.82-0.92) | 0.86  (0.81-0.92) |
| **WC, cm** | 0.99  (0.96-1.01) | 0.99  (0.96-1.02) | 0.98  (0.95-1.01) | 0.99  (0.96-1.02) | 0.99  (0.96-1.02) |
| **MUAC, cm** | 0.86  (0.78-0.95) | 0.90  (0.82-1.00) | 0.83  (0.74-0.93) | 0.87  (0.78-0.96) | 0.87  (0.78-0.96) |
| **MUAMC, cm** | 0.91  (0.81-1.03) | 0.94  (0.85-1.04) | 0.90  (0.80-1.02) | 0.92  (0.82-1.04) | 0.91  (0.81-1.02) |
| **SFT, mm** | 0.96  (0.91-1.00) | 0.97  (0.93-1.01) | 0.95  (0.91-1.00) | 0.96  (0.92-1.01) | 0.96  (0.92-1.01) |

ALM**_BIA_**, appendicular lean mass assessed by bioelectrical impedance analysis; ALMI**_BIA_**, appendicular lean mass index assessed by bioelectrical impedance analysis; BMI, body mass index; HGS, handgrip strength; MUAC, mid-upper arm circumference; MUAMC, mid-upper arm muscle circumference; PA, phase angle; SFT, skinfold triceps; WC, waist circumference. HGS: Maximum measure of six measurements (three at each side) was applied, irrespective of side dominance. No diagnosis of sarcopenia considered reference, Sex: male considered reference, diabetes: no diagnosis considered a reference, no dialysis treatment considered reference.
